# Reply to Comment on Oldenburg, M., Jensen, H.J. Stress and Strain among Seafarers Related to the Occupational Groups. *Int. J. Environ. Res. Public Health 2019*, doi:10.3390/ijerph16071153

**DOI:** 10.3390/ijerph17062137

**Published:** 2020-03-23

**Authors:** Marcus Oldenburg, Hans-Joachim Jensen

**Affiliations:** 1 Institute for Occupational and Maritime Medicine Hamburg (ZfAM), University Medical Center Hamburg-Eppendorf (UKE), 20459 Hamburg, Germany; 2 Maritime Centre, University of Applied Sciences, 24943 Flensburg, Germany; hans_joachim-jensen@t-online.de

In respect of the above mentioned manuscript [1], the authors received some comments that we would like to address. The reviewer criticized the fact that no standardized questionnaire, such as the Job–Demand–Control support model by Karasek, or questionnaires about boredom had been applied in our study. This was, however, not the aim of the present survey. As mentioned in the manuscript, our study aimed to assess the stress and psychophysical strain of seafarers using objective methods. Several previous studies have already dealt with the subjective assessment of stress and strain experienced on board seafaring vessels, many of them using standardized instruments. Additionally, only a few of them focused on objective parameters, such as the armband monitor or the Polar watch used in our study. In order to roughly assess the extent of the seafarers’ subjective stress, the sailors were only briefly asked about their subjective stress experiences due to job-related physical or mental impacts, in accordance with ISO 10075-1:2017 [2] and structured as a five-item Likert scale.

Generally, it must be agreed that standardized instruments are preferred for the evaluation of subjective attitudes. In specific working surroundings (like those in seafaring), however, there are numerous impacts (e.g., permanent noise, vibration, ship movements over a 24 h day, separation from family and friends for several months, multicultural crews, extraordinarily high time pressure during port stays, objectively measured sleep deficiency, i.e., only 5 h of sleep per day). In total, the complexity of the working and living situation on board ships is unique and not comparable with working environments ashore. This was the main reason for the development of a seafaring-specific questionnaire that has repeatedly been used and cited in previously published studies on seafaring stressors [3,4]. Moreover, the questionnaire was developed for a multicultural crew with different levels of English and varied educational backgrounds.

Furthermore, the reviewer recommended discussing the problem of boredom on board. Boredom is undoubtedly an important aspect in the working routine on board. However, this topic is only one of several other mental stressors among the crew (e.g., isolation, hierarchical structures, time pressure, kinetosis, a lack of social support on board, long periods of separation from family, etc.) and was not the focus of the present manuscript.

Additionally, boredom on vessels needs to be discussed controversially. Firstly, it is likely that boredom on board depends on the shipping route; in the present study, 22 sea voyages were conducted in the North/Baltic Sea area (including the English Channel) or in a comparable coastal operation. These voyages are characterized by a high port frequency leading to only short sea passages that are unlikely to cause boredom. On the other hand, the crews of worldwide-operating vessels (who were not included in our study) are more prone to developing boredom at sea.

Secondly, the different activity profiles of the various working groups on board should be considered. As previously published, the work diversity differs, especially among nautical officers and deck ratings, with a variety of requirements between the voyage periods [5]. This is one reason why in, the present on-board investigation, a distinction is made between the three occupational groups of nautical officers, deck ratings and engine room personnel. As the occupational groups on board exhibit differences in the diversity of their work and also their educational level, it is assumed that the topic of boredom is strongly associated with the working group as a dependent parameter.

Finally, we agree with the reviewer that further studies on the job-related stressors of seafarers (including boredom) on board are recommended.
